# The prevalence and healing of apical periodontitis in patients with autoimmune diseases

**DOI:** 10.1111/iej.14214

**Published:** 2025-03-02

**Authors:** Francesco Mannocci, Garrit Koller, Sushmita Ravindran

**Affiliations:** ^1^ Department of Endodontics, Faculty of Dentistry, Oral and Craniofacial Sciences King's College London London UK; ^2^ Department of Restorative Dentistry Universiti Sains Islam Malaysia Nilai Malaysia

**Keywords:** apical periodontitis, autoimmune disease, healing, prevalence

## Abstract

Apical periodontitis (AP) is a common and clinically significant oral health condition, associated with an inflammatory response to infections within the root canal system. As patients retain natural teeth for longer, managing AP becomes more complex. Whilst generally effective, endodontic treatment outcomes can vary considerably in individuals with systemic health conditions, such as autoimmune diseases. The intersection of systemic inflammation, immune dysfunction and pharmacological treatments of the different diseases raises important questions about how autoimmune diseases influence AP prevalence and healing. This article examines current evidence on this interplay, its clinical implications and the need for tailored endodontic approaches in patients with autoimmune diseases.

## INTRODUCTION

Apical periodontitis (AP) is primarily an inflammatory response to contain infections spreading from the root canal space to the periapical tissues (Kakehashi et al., 1965). Systemic conditions may modulate AP prevalence and outcomes, necessitating tailored approaches in clinical management. The host response, governed by both innate and adaptive immunity, is central to the pathogenesis of AP (Nair, 2004). Understanding factors affecting the host immune response in apical periodontitis is of growing importance, especially as more patients with autoimmune conditions undergo endodontic treatments.

Autoimmune conditions are characterized by chronic systemic inflammation and immune dysregulation, which may alter susceptibility to infections and impair tissue healing. These pathophysiological changes raise important questions regarding their influence on AP.

Several studies have investigated the association between autoimmune diseases and oral health. Evidence suggests that patients with rheumatoid arthritis (RA) and inflammatory bowel disease (IBD) exhibit higher rates of apical periodontitis, potentially due to increased infection susceptibility and impaired healing mechanisms (Karataş et al., 2020; Piras et al., 2017). While systemic factors are undoubtedly influential, the precise impact of immunomodulatory and immunosuppressive therapies on AP prevalence and healing remains poorly understood.

Further complicating this relationship is the role of coexisting factors, such as elevated caries risk, often reflected in higher DMFT values among many patient cohorts with autoimmune diseases (Allihaibi et al., 2023; Barros et al., 2020; Martinez‐Martinez et al., 2019; Poyato‐Borrego et al., 2020; Zhang et al., 2020), suggesting that higher caries levels may be more relevant than their immunological condition in contributing to the development of more AP lesions. The overlapping influence of systemic and oral conditions underscores the need for an improved understanding of AP in these populations.

This article examines current knowledge of the prevalence and healing dynamics of AP in patients with autoimmune diseases, emphasising the immunological and pharmacological influences on its clinical course.

## AUTOIMMUNE DISEASES AND THEIR INFLUENCE ON APICAL PERIODONTITIS

Patients with autoimmune conditions, such as rheumatoid arthritis (RA), systemic lupus erythematosus (SLE) and inflammatory bowel disease (IBD), often exhibit chronic systemic inflammation and immune dysregulation. These factors can significantly influence the clinical course of AP. Chronic inflammation, driven by persistently elevated levels of proinflammatory cytokines, such as tumour necrosis factor‐alpha (TNF‐α), interleukin‐1 (IL‐1) and interleukin‐6 (IL‐6), are central to the pathogenesis of many autoimmune diseases. These same cytokines are implicated in the periapical tissue destruction observed in AP, suggesting shared mechanisms of tissue damage (Guo et al., 2018). Similar mechanisms are seen in other autoimmune diseases where hyper‐activated immune pathways perpetuate tissue damage, hindering the effective resolution of AP lesions.

In RA, overproduction of TNF‐α and IL‐1 contributes to joint destruction but may also exacerbate AP by promoting periapical bone resorption and delaying healing. Similar inflammatory pathways are observed in IBD and SLE, where dysregulated immune responses hinder the effective resolution of AP lesions. Autoimmune reactions, such as those associated with the presence of anti‐citrullinated protein antibodies (ACPAs), have also been detected in periapical granulomas (Martos et al., 2023; Wichnieski et al., 2019).

Conventional and more recently developed intracanal medications have, to a certain extent, immunomodulatory effects on periapical tissue inflammation (Hussein & Kishen, 2022). However, a growing body of literature has examined the impact of the systemic use of immunosuppressants and immunomodulators on AP.

Anti‐inflammatory and Immunosuppressive therapies used to manage autoimmune diseases may affect the clinical course of AP. Corticosteroids, while effective at suppressing inflammation, may impair microbial defence mechanisms, potentially worsening AP outcomes (do Nascimento et al., 2022). Conversely, biologic DMARDs (bDMARDs) that target specific inflammatory pathways have shown mixed effects on AP.

Some studies indicate that (bDMARD) may not significantly alter the prevalence of AP compared to patients not on these medications (Allihaibi et al., 2023). In contrast, others showed that patients affected by autoimmune and alcohol‐related liver diseases (ALDs) undergoing treatment with immune suppressors (often associated with immune modulators) exhibited a lower prevalence of AP (Ideo et al., 2022, 2024).

This suggests that while autoimmune diseases may predispose patients to develop AP more frequently, the medications used to treat these conditions might not necessarily confer additional risk or benefit regarding AP.

## EVIDENCE OF ASSOCIATION

In a retrospective study, Jalali et al. (2017) found no significant difference in the prevalence of apical periodontitis between patients with and without rheumatoid arthritis.

Analogies have been drawn between the autoimmune components of marginal and apical periodontitis, as both diseases share similarities in the bacterial flora that cause them, are mediated by proinflammatory cytokines, and result in tissue destruction (Martos et al., 2023). The relationship between autoimmune diseases like rheumatoid arthritis, their treatment, and periodontal disease has been extensively studied.

A recent systematic review (Petit et al., 2024) failed to find significant improvement in periodontal conditions of RA patients treated with conventional synthetic DMARDs (csDMARDs) reported worse gingival inflammation associated with treatment using TNF inhibitors combined with methotrexate, and noted some improvement in the periodontal condition of patients treated with bDMARDs when the follow‐up was limited to 6 months; the same drugs had a negligible effect in longer‐term studies. The results regarding tooth retention were inconclusive, with some studies and drugs being associated with better and others with worse tooth retention. Several periodontal disease studies were interventional (Kobayashi et al., 2014, 2015); however, similar studies have not been attempted in endodontics.

The somewhat positive effects of bDMARDs on periodontal conditions are unlikely to be replicated in apical periodontitis. In both treated and untreated periodontal disease (Goodson et al., 1982; Haffajee & Socransky, 1986), there may be periods of slow progress and periods in which the destructive processes accelerate; however, the inflammatory destruction of the periodontium progresses somewhat steadily over time and this progress is easily traceable. There is an almost direct correlation between the progression of attachment loss and tooth loss. It is, therefore, understandable that anti‐inflammatory drugs taken for many years or applied locally during the phases of acceleration of the periodontal destructive processes may slow the progression of attachment loss and potentially increase tooth retention.

It has been argued that excessive bone resorption caused by apical periodontitis not responding to conventional endodontic treatment may also lead to the need for apical surgery or tooth extraction, and this would justify the need to develop immunotherapeutic strategies to mitigate periapical tissue destruction (Wen et al., 2024).

However, the progression of untreated and treated AP does not seem to be steady, and the probability of developing acute apical periodontitis and its complications leading to tooth loss is not linearly correlated with the size of apical radiolucencies. Furthermore, the progression of AP is much more difficult to measure quantitatively over time than that of marginal periodontitis, as in most cases, its presence is either detected incidentally when periapical radiographs are taken for other reasons or diagnosed when the tooth becomes symptomatic.

**FIGURE 1:**
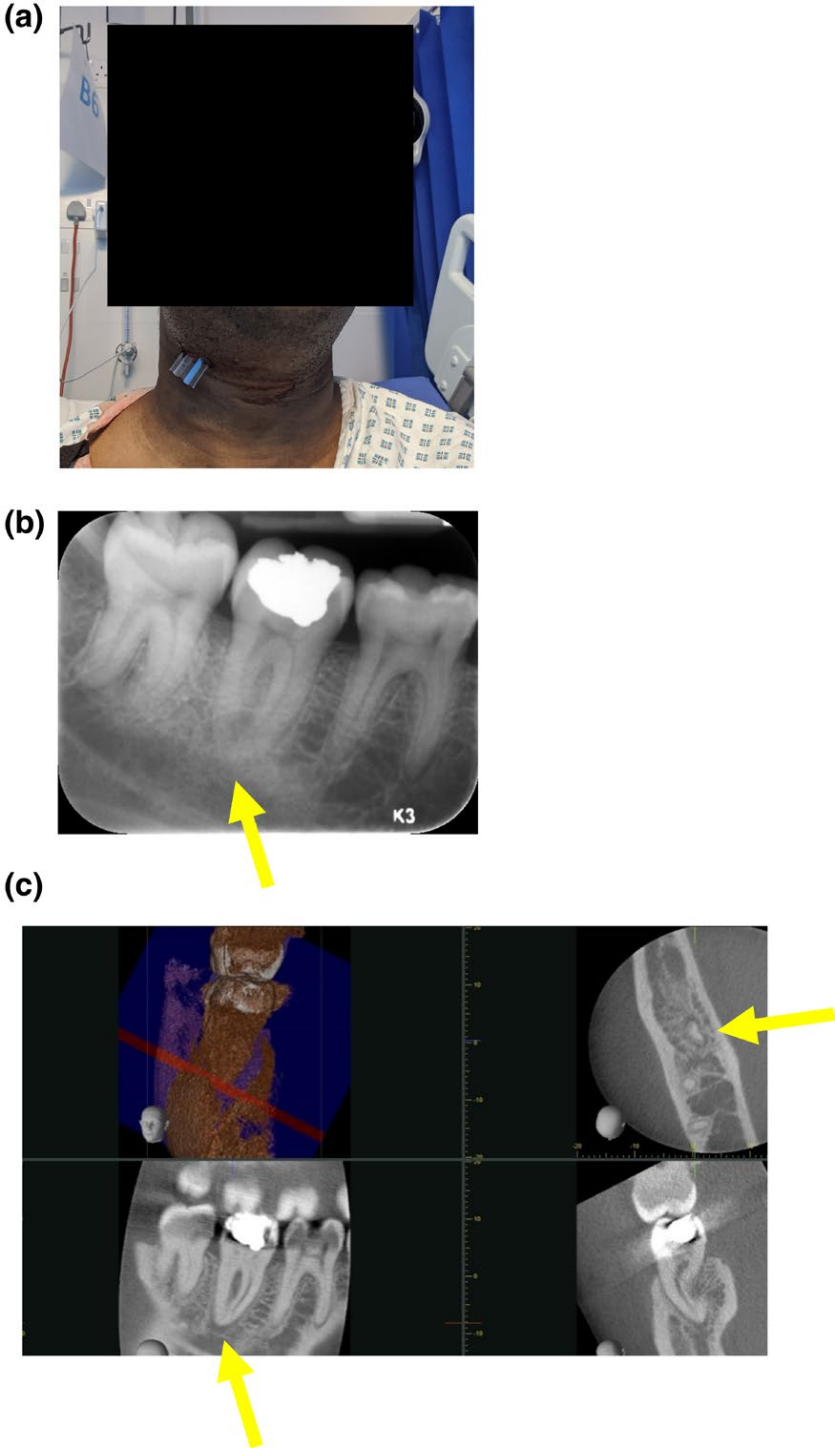
(a) The pulp necrosis of the lower right second molar (LR7) has caused a large abscess with cellulitis requiring extra‐oral drainage. (b, c) The periapical radiograph does not show any obvious radiolucency and the CBCT scan shows the presence of a 2 mm apical radiolucency associated with the LR7 (indicated by the yellow arrow).

Teeth with larger apical radiolucencies are more likely to become symptomatic and require treatment or extraction over time than teeth with small lesions; still, eventually, many teeth with very small periapical lesions may become acutely symptomatic, leading to root canal treatment/re‐treatment/apical surgery or extraction of the tooth (Figure 1). In many cases, pulpal necrosis may remain clinically silent, showing no radiographic sign of progression for some time and displaying evidence of disease only after many years.

It could be argued that long‐term systemic immunomodulatory strategies are likely to have a limited effect on AP and tooth loss caused by AP during periods of no detectable progression. In fact, patients taking immunomodulators (Allihaibi et al., 2023; Ideo et al., 2022) do not appear to have an increased tooth retention rate when compared with the controls.

Endodontic medications and sealers containing immunomodulators are also likely to have a limited effect on apical periodontitis as, contrary to periodontal medications, they cannot be repetitively applied to the affected tissues in the recurrent phases of disease progression and are likely to be active only for a short time following their application into the root canal space.

## CLINICAL AND THERAPEUTIC IMPLICATIONS

Endodontic treatment in patients with autoimmune diseases requires careful consideration of both the disease and the disease‐associated pharmacotherapy. It remains unclear whether the increased prevalence of apical periodontitis in these patients is caused by the immune conditions or by higher levels of caries, as shown by higher DMFT values, or both; however, the higher prevalence of AP in this patient group suggests a need for more frequent monitoring and perhaps earlier intervention to prevent the progression of lesions. The co‐existence with autoimmune diseases of systemic conditions, such as diabetes, can further complicate endodontic healing (Segura‐Egea et al., 2016).

Moreover, clinicians must account for the potential impact of immunosuppressants and immunomodulators on the healing trajectory. Patients on long‐term corticosteroid or biologic DMARD therapy may also require prolonged follow‐up periods, and insights from these will elucidate the impact of these drugs, if any, on the outcome of root canal treatments, re‐treatments and surgical endodontics. Therefore, a multidisciplinary approach, involving close collaboration with other specialists, may be necessary to optimise patient outcomes.

## CONCLUSION

The prevalence of apical periodontitis in patients with autoimmune diseases and those treated with immunosuppressants and immunomodulators underscores the complex interplay between systemic health, medication, and endodontic outcomes. While the evidence suggests a higher incidence of AP in these patients, the role of immunosuppressive therapies remains equivocal, with some studies pointing to a limited impact on AP development and healing. Future research should aim to elucidate the precise mechanisms by which autoimmune diseases and their treatments influence AP, with the goal of developing more effective therapies for AP.

## AUTHOR CONTRIBUTION

Francesco Mannocci, Garrit Koller, Sushmita Ravindran: conceptualization and writing

## CONFLICT OF INTEREST STATEMENT

The authors declare that they have no conflict of interest.

## Data Availability

Data sharing is not applicable to this article as no new data were created or analyzed in this study.
